# Complete remission of chemo‐refractory multiple‐metastatic upper tract urothelial carcinoma by autologous formalin‐fixed tumor vaccine

**DOI:** 10.1002/ccr3.1179

**Published:** 2017-09-15

**Authors:** Tatsu Miyoshi, Takeshi Kashiwabara, Atsuko Asahi, Tatsuji Kataoka, Takashi Maruyama, Rika Okada, Yoji Uemae, Tadao Ohno

**Affiliations:** ^1^ Ginza‐Namiki‐Dori Clinic Chuo‐ku, Tokyo Japan; ^2^ Saku Central Hospital Saku City Nagano Pref. Japan; ^3^ Department of Neurosurgery Tokyo Women's Medical University Shinjuku‐ku, Tokyo Japan; ^4^ Cell‐Medicine, Inc. Tsukuba Science City Ibaraki Japan

**Keywords:** Cancer vaccine, immunotherapy, monotherapy, urothelial carcinoma

## Abstract

A patient with chemo‐refractory multiple‐metastatic upper tract urothelial carcinoma (UTUC) treated by monotherapy with autologous formalin‐fixed tumor vaccine (AFTV) resulted in complete remission of the lung and para‐aortic lymph node metastases (ongoing >3 years after AFTV). The tumor was immunohistologically negative for PD‐L1. AFTV will be an attractive treatment option.

## Introduction

Upper tract urothelial carcinoma (UTUC) is a rare disease, accounting for 5% of urothelial malignancies 1, 2. Radical nephroureterectomy (RNU) with the excision of the bladder cuff is considered the surgical standard of treatment for nonmetastatic UTUC. However, about 30% of patients develop recurrent disease after RNU. The 1‐ and 3‐year survival rates of patients after RNU are 39.5% and 9.4%, respectively 1. Here, we report on a case of multiple‐metastatic, but defective PD‐L1 expression, UTUC that was successfully treated by monotherapy with autologous formalin‐fixed tumor vaccine (AFTV) after failed RNU and adjuvant chemotherapy.

## Case Report

**Figure 1:**
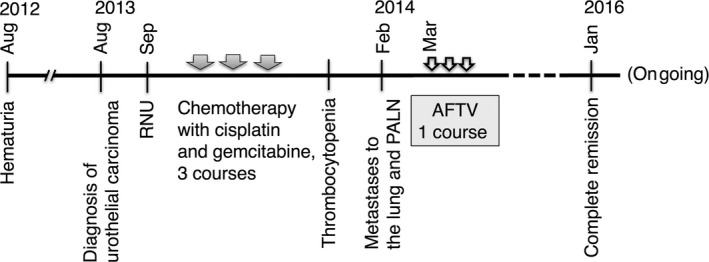
Patient's clinical course. RNU, Radical nephroureterectomy; PALN, para‐aortic lymph node; AFTV, autologous formalin‐fixed tumor vaccine.

**Figure 2:**
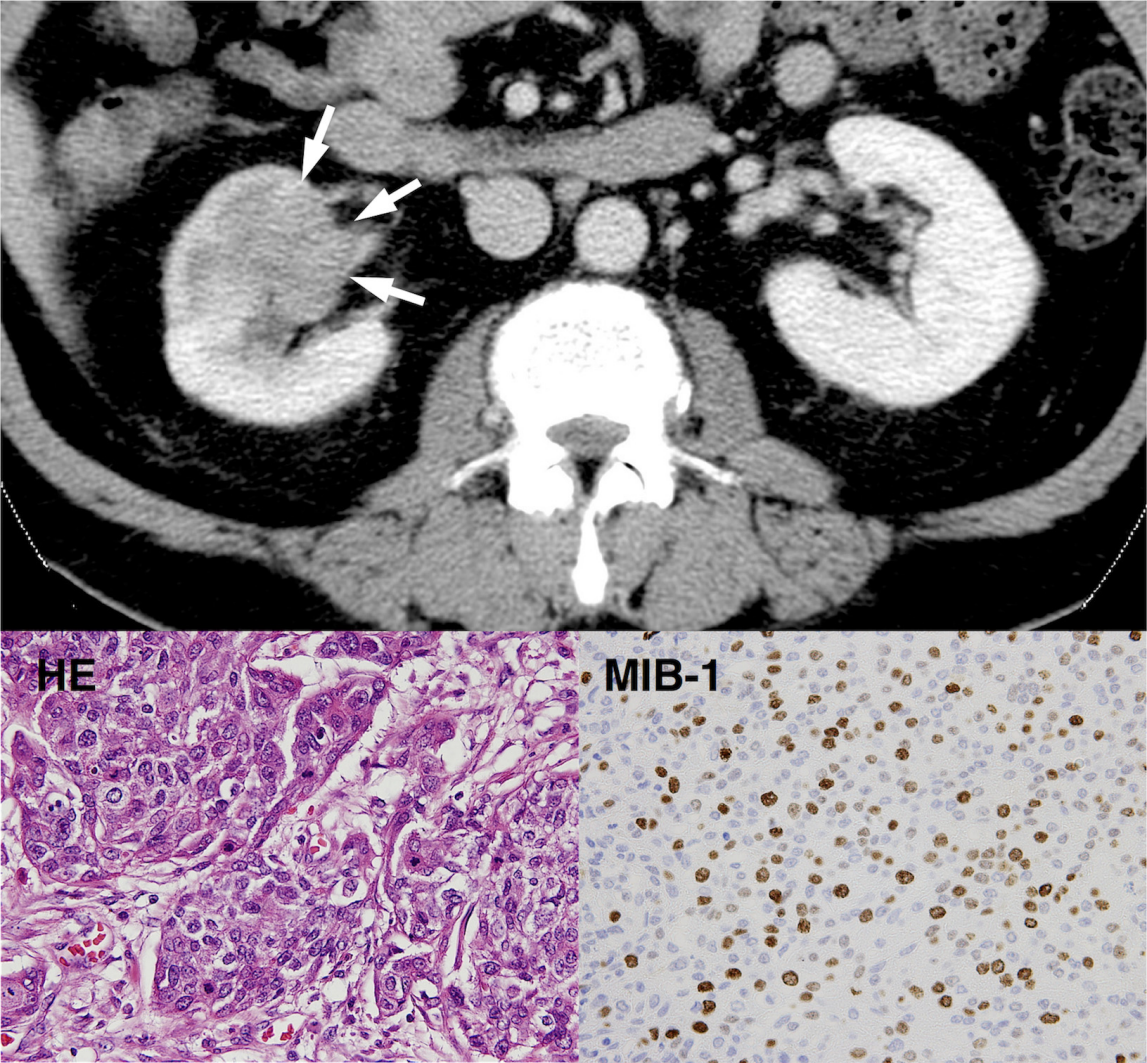
CT scan showed a walnut‐size (diameter of approximately 40 mm) abnormal shadow in the right kidney (arrow head). Lower left, H&E staining of resected specimen of the present case revealing an infiltrating urothelial carcinoma with hyperchromatic nuclei and a high grade of atypism (magnification, × 400). Lower right, highly positive for MIB‐1, an index of the cell proliferation.

A 62‐year‐old male was hospitalized for the evaluation of hematuria in August 2012. No tumor was detected in the urinary system by ultrasonography, and urine cytology was class II (patient's clinical course was illustrated in Fig. 1). A year later in August 2013, both ultrasonography and a computed‐tomography (CT) scan revealed a walnut‐sized abnormal mass in the right kidney that was diagnosed as urothelial carcinoma (Fig. 2). He underwent RNU in September 2013. The surgically resected tumor of the right kidney was a gray‐white colored, solid, relatively well demarcated, and 50 × 50 × 40 mm in size. The tumor had infiltrated the renal parenchyma but had not reached the kidney surface. Histologically, the tumor was diagnosed as a high‐risk urothelial carcinoma (p‐T3N0) composed of cancer cells with hyperchromatic nuclei and a high grade of nuclear atypia. The tumor showed relatively high positivity for MIB‐1 (40%), an index of cell proliferation (Fig. 2, lower right).

As the pathological findings indicated a poor prognosis, adjuvant chemotherapy was performed with cisplatin (70 mg/m^2^, day 2) and gemcitabine (1000 mg/m^2^, day 1, 8, 15) in a 3‐week regimen. However, severe thrombocytopenia developed (CTCAE grade 4). The regimen was stopped after completion of three courses of the chemotherapy. Subsequently, metastases to the lung and para‐aortic lymph node (PALN) were revealed by a CT scan in February 2014, only 5 months after RNU. The patient did not wish to receive further aggressive chemotherapy. We introduced AFTV according to strong request by the patient.

**Figure 3:**
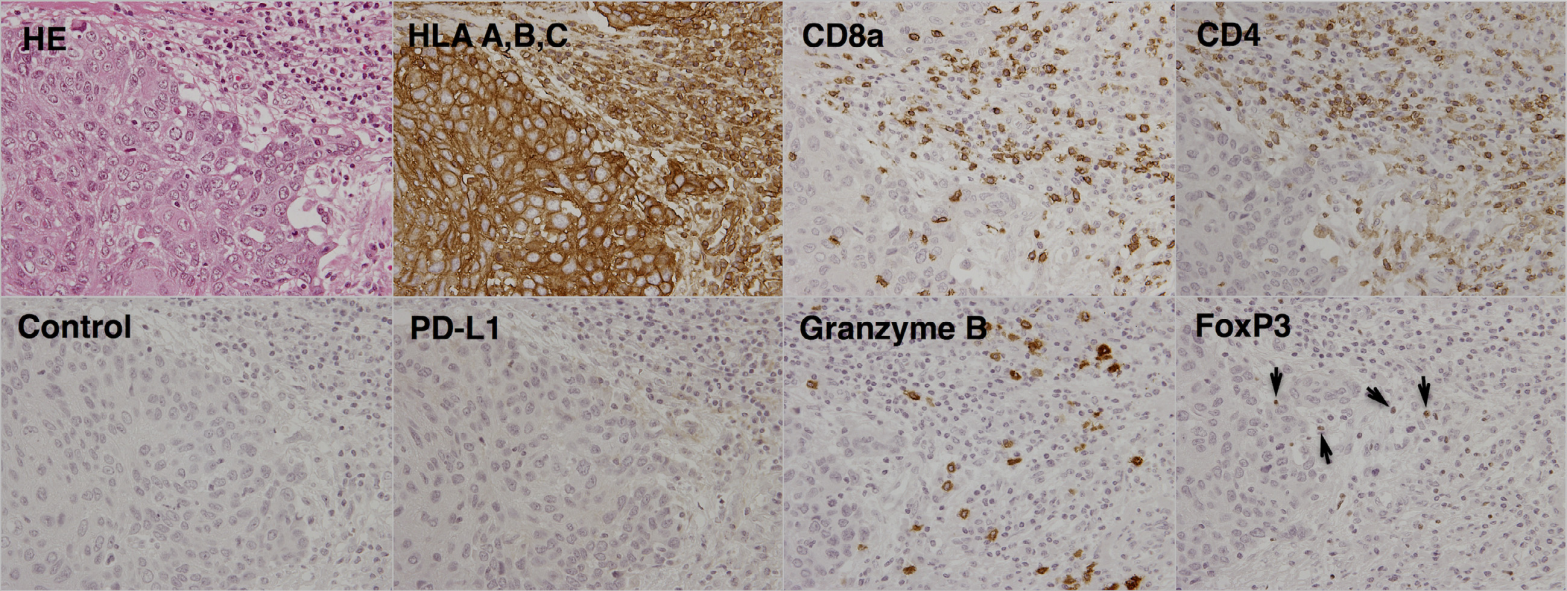
Immunohistochemically, HLA A, B, C (MHC‐class I) were strongly expressed on the tumor cells. CD8a, CD4, and granzyme B were stained in the immune cells in the tumor. FoxP3 was expressed (arrow heads), but no PD‐L1 expression was detected on the tumor cells.

In order to observe the immune status in the tumor tissue before the AFTV administration, the histopathological expression of HLA A, B, C (MHC‐class I), CD8a, CD4, programmed death‐ligand 1 (PD‐L1), granzyme B, and forkhead box P3 (FoxP3) were examined. MHC‐class I was strongly expressed on the tumor cells as well as CD8a, CD4 (Fig. 3, upper side), and granzyme B (Fig. 3, lower side), which were stained in immune cells infiltrating the tumor. Also, the expression of FoxP3 was found (arrow heads in Fig. 3, lower right), but PD‐L1 expression was not observed on the tumor cells (Fig. 3, lower left).

**Figure 4:**
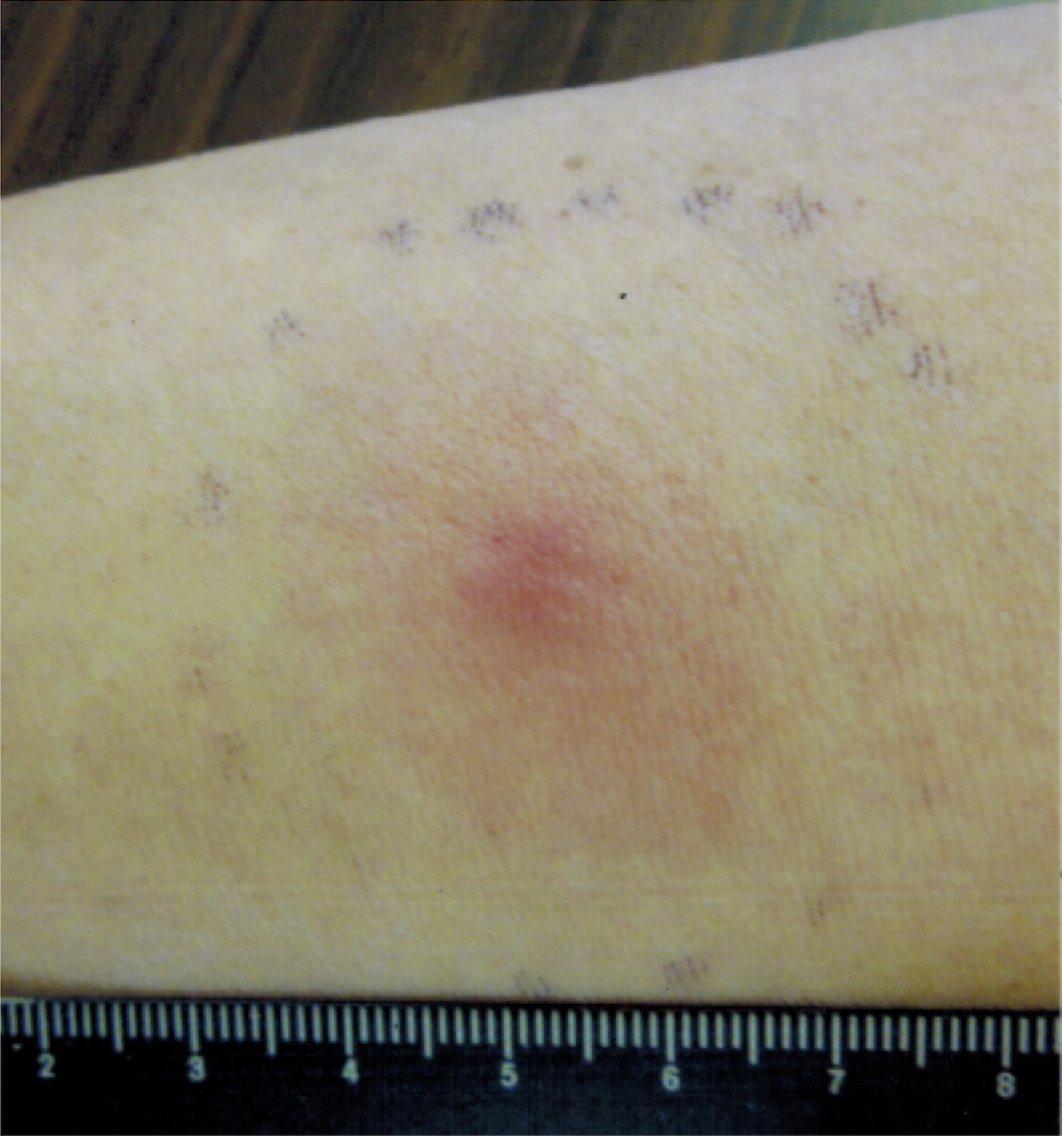
Delayed‐type hypersensitivity response to autologous formalin‐fixed tumor fragments (without immunoadjuvant) became positive (30 × 30 mm erythema and 10 × 10 mm induration) after the third AFTV injection.

With approval from the ethical authority of Ginza‐Namiki‐Dori Clinic and the informed consent of the patient, we prepared AFTV as previously reported 3. We used 3.4 g of autologous formalin‐fixed carcinoma tissue, which was presumed to include tumor‐associated antigens. The vaccine was intradermally injected into the patient's upper arm once a week, for 3 weeks, after the middle of March 2014. To evaluate the cell‐mediated immunity status of the patients, the delayed‐type hypersensitivity (DTH) response was tested 2 weeks after the third AFTV injection (formalin‐fixed tumor tissue fragments containing no immunoadjuvant), which was positive (30 × 30 mm erythema and 7 × 7 mm induration) (Fig. 4). During this treatment, no adverse effects were observed except for a slight rash (CTCAE grade 1), appeared at local injection sites of AFTV, which gradually disappeared over a few months.

**Figure 5:**
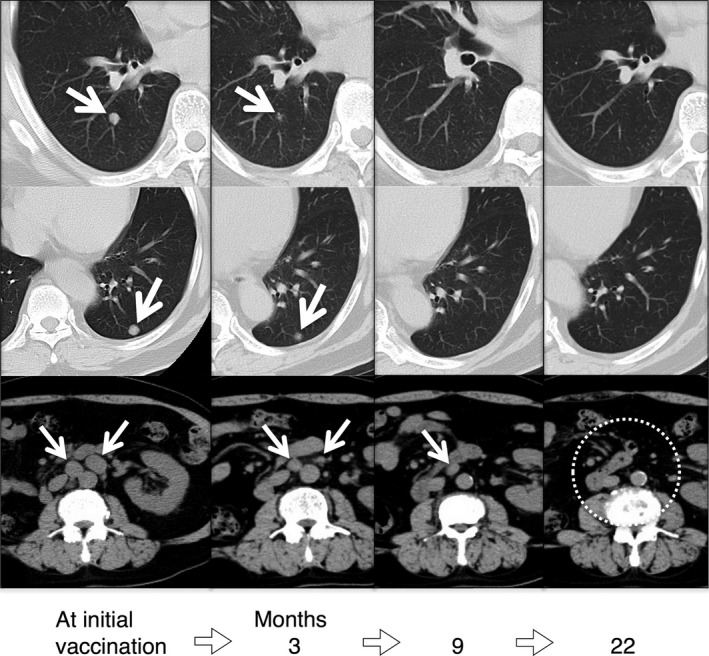
CT images in the metastasized lung and para‐aortic lymph node lesions. Multiple metastases to lung and para‐aortic lymph nodes were detected at the initial vaccination with AFTV. Three months after the first vaccination with AFTV, all of the metastatic lesions showed a tendency to decrease in size. Nine months after the first vaccination with AFTV, disappearance of the metastases in lung and further reduction of para‐aortic lymph node metastases were detected. Twenty‐two months later, all metastases disappeared from the image.

Serial CT scans are shown in Figure 5. Multiple metastases to lung and PALN were detected at the time of the initial vaccination. However, 3 months later, they showed a tendency to decrease in size. Moreover, 9 months later, a complete remission of the lung metastases and further reduction of PALN metastases were detected. Twenty‐two months later, all metastases had disappeared on CT imaging. Since the first vaccination, 36 months have passed and no recurrence has been found. During this period, no other treatment including any immune checkpoint inhibitors has been provided.

## Discussion

The prognosis for metastatic UTUC after RNU is very poor; 1‐ and 3‐year overall survival rates are 39.5% and 9.4%, respectively, with median overall survival time 10 months 1. Recurrence is inevitable and there is no effective treatment. The present case was refractory to conventional therapy (RNU and platinum‐based chemotherapy), and the patient strongly desired new treatments for the lung and PALN metastases. The patient showed complete response to AFTV with no remarkable adverse events.

The immunohistochemical findings (Fig. 3), expression of MHC‐class I on tumor cells and CD8a and granzyme B on immune cells within the tumor, suggest that an active cytotoxic immune response may be inducible against tumor cells. Furthermore, PD‐L1 was negative on the tumor cells, suggesting that the immune checkpoint inhibition by PD‐L1 may not occur in the present case. As a result, the FoxP3‐positive cells observed in the tumor (generally believed to include regulatory T cells) did not exhibit a strong inhibition of the killing action of the tumor‐infiltrating CTLs. As shown in Figure 4, the positive DTH response in the present case suggests that CTLs were induced in vivo following vaccination.

We have reported previously on the prophylactic effect of AFTV (one course, i.e., three vaccinations as mentioned for the present case, which is sufficient to induce a DTH response) to suppress the frequent recurrence of hepatocellular carcinoma (HCC) in a randomized study 4, in a re‐recurrent case of HCC, in which AFTV induced glypican‐3‐specific cytotoxic T lymphocytes (CTL) 5, and a case of multiple‐recurrent HCC that had previously been treated 29 times with various modalities, including three laparotomies 6. A typical therapeutic effect of AFTV was also observed in the eradication of bone‐metastasized mammary carcinoma 7. More importantly, we are studying the clinical effects of AFTV in patients with newly diagnosed glioblastoma multiforme (GBM) in which complete resection is not possible. While the present standard therapy for newly diagnosed GBM provides a median overall survival time (mOS) of 14.6 months 8, the mOS reached 22.2 months in one of our clinical trials 9. Patients who showed a positive DTH response to autologous formalin‐fixed GBM fragments (without immunoadjuvant) following vaccination with AFTV survived longer than those showing a negative DTH response (*P* = 0.0071, log‐rank test) 9. Thus, we conclude that AFTV is able to provide both patient‐specific and tumor‐specific antigens.

Although, obviously, further investigation in a larger scale clinical trial is required to validate our results, the present case offers an intriguing first glimpse of the potential contribution of immunotherapy with AFTV.

## Conclusion

Treatment with cancer vaccine such as AFTV will probably be suitable for chemo‐refractory metastatic UTUC, if the tumor is PD‐L1‐negative.

## Conflict of Interest

None declared.

## Authorship

TM, the TKs, AA, and TM: carried out vaccination with AFTV. YU and TO: prepared AFTV. RO: coordinated the treatment courses. All authors read and approved the final manuscript.
